# Eliciting and Understanding Primary Care and Specialist Mental Models of Cirrhosis Care: A Cognitive Task Analysis Study

**DOI:** 10.1155/2021/5582297

**Published:** 2021-06-15

**Authors:** Tanya Barber, Lynn Toon, Puneeta Tandon, Lee A. Green

**Affiliations:** ^1^Department of Family Medicine, University of Alberta, T6G 2T4, Edmonton, AB, Canada; ^2^Accelerating Change Transformations Team, Alberta Medical Association, T5N 3Y8, Edmonton, AB, Canada; ^3^Division of Gastroenterology (Liver Unit), Zeidler Ledcor Centre, T6G 2X8, Edmonton, AB, Canada; ^4^Faculty of Medicine, University of Alberta, T6G 2R7, Edmonton, AB, Canada; ^5^Kaye Edmonton Clinic, T6G 1Z1, Edmonton, AB, Canada

## Abstract

**Background:**

Gaps in coordination and transitions of care for liver cirrhosis contribute to high rates of hospital readmissions and inadequate quality of care. Understanding the differences in the mental models held by specialty and primary care physicians may help to identify the root causes of problems in the coordination of cirrhosis care.

**Aim:**

To compare and identify differences in the mental models of cirrhosis care held by primary and specialty care physicians and nurse practitioners that may be addressed to improve coordination and transitions.

**Methods:**

Cross-sectional formal elicitation of mental models using Cognitive Task Analysis. Purposive and chain-referral sampling to select family physicians (*n* = 8), specialists (*n* = 9), and cirrhosis-dedicated nurse practitioners (*n* = 2) across Alberta.

**Results:**

Family physicians do not maintain rich mental models of cirrhosis care. They see cirrhosis patients relatively infrequently, rebuilding their mental models when required (knowledge on demand). They have reactive and patient-need-focused, rather than proactive and system-of-care, mental models. Specialists' mental models are rich but vary widely between patient-centered and task-centered and in the degree to which they incorporate responsibility for addressing system gaps. Nurse practitioners hold patient-centered mental models like specialists but take responsibility for addressing gaps in the system.

**Conclusions:**

Improving the coordination of cirrhosis care will require infrastructure to design care pathways and work processes that will support family physicians' knowledge-on-demand needs, facilitate primary care-specialist relationships, and deliberately work toward building a shared mental model of responsibilities for addressing medical care and social determinants of health.

## 1. Introduction

Liver cirrhosis is a leading cause of morbidity and premature mortality in patients with a digestive disease [1, 2]. The decompensated stage of cirrhosis is defined by the presence of cirrhosis complications including ascites, hepatic encephalopathy, and variceal hemorrhage [3, 4]. In addition to these medical complications, patients across both compensated and decompensated stages have additional complexity due to comorbid physical frailty, addiction, psychological distress, socioeconomic instability [1, 5, 6], and high suffering [7]. A minority of patients (∼one-third) receives best-practice care around cirrhosis complications, broader health needs (e.g., alcohol screening) or care transitions (e.g., follow-up visit booked at hospital discharge) [8]. These quality gaps contribute to unacceptable rates of hospitalization, rehospitalization (53% at 90 days), and high costs of care [9, 10]. Recent data in cirrhosis supports the beneficial impact of shared care across multidisciplinary providers (specialty care, primary care, and advance practice providers such as nurse practitioners) as a key strategy to improve quality of care and clinical outcomes [11–13]. However, research has also demonstrated differences in specialist and general practitioner care, and a lack of clarity in roles and responsibilities between these two groups [14, 15]. Primary care physicians have also experienced a reluctance to drive cirrhosis care when they lack expertise or confidence in this area [14, 16, 17]. A deeper understanding of why primary care and specialist physicians approach cirrhosis care differently, vary in their confidence levels, and perceive their roles and responsibilities is needed [18].

The Cirrhosis Care Alberta Program (CCAB) is a 4-year multicomponent quality improvement initiative that aims to improve quality of care, reduce acute care utilization, and provide a high level of care satisfaction to both patients and health care providers within the province of Alberta [8]. Recognizing the impact of shared care on outcomes, one of CCAB's goals is to better understand and improve role clarity, coordination, and integration between primary care and specialty services. This aligns with international goals for transitions of care [19] as well as Alberta's own health system goals [20–23].

Commonly, integration and coordination of care have been approached as a logistical issue, with solutions including tools and measures that set out to improve communication between primary and specialty care. Although these tools have value, they need to suit both specialist and primary care physicians' workflow which often leads to deficits in uptake and continued use [15]. In addition, they do not directly assess the root of the issue, which is that family and specialty physicians think about and approach cirrhosis care quite differently (e.g., they have different mental models of cirrhosis care) [15, 24, 25].

Mental models are more than sets of beliefs and values; they are dynamic structures. Our mental models determine what we pay attention to; what options and possibilities we consider; and how we make sense of events and experiences, solve problems, formulate judgments, and ultimately make decisions and act. Understanding mental models is an important aspect of transformation within primary care [25–27].

Therefore, we conducted a province-wide study to compare the mental models of cirrhosis care held by specialty and primary care physicians and nurse practitioners. Our goal was to identify differences in mental models that CCAB and other programmatic initiatives could address to improve transitions and coordination between primary and specialty care.

## 2. Materials and Methods

This was a cross-sectional formal elicitation of mental models using a Cognitive Task Analysis (CTA) technique called the Knowledge Audit [28]. CTA is a set of highly structured qualitative techniques used to elicit the cognitive activities individuals perform to accomplish tasks in real-world settings. It has produced an accurate understanding of knowledge work in many high-stakes areas such as aviation, firefighting, and Intensive Care Units [28]. This study received Research Ethics Board approval from the University of Alberta (Pro00091294).

### 2.1. Participants

Chain-referral sampling was used [29] to selectively recruit family physicians (*n* = 8) who saw small numbers, typical for ordinary practice, of cirrhosis patients, and specialists (*n* = 9) with high content knowledge who saw many cirrhosis patients. Family physicians with unusual interest and expertise in cirrhosis were not included. Two nurse practitioners working in cirrhosis care clinics were added to the sample because multiple physician interviews mentioned coordinating care with them, suggesting their perspective would be important. The participants varied in terms of gender, age, years of practicing, and geographic location within Alberta.  Table 1 provides details of participant demographics.

We posted notices about the study in the Alberta Medical Association's (AMA) provincial newsletter and on their website, asking physicians to contact us if interested in participating. Those interviewed were asked to nominate other physicians or nurse practitioners they thought would be interested in participating. Over a period of six months, 19 participants were recruited and interviewed. No participants withdrew from the study. Of the 19 participants, 7 self-identified through notices, and 10 were nominated by other providers. The 2 NPs were nominated by CCAB.

### 2.2. Data Collection

Team members from the AMA-Accelerating Change Transformation Team (ACTT), an interviewer and a notetaker, trained in CTA, conducted one-hour interviews with participants. Most interviews were conducted in person; however, two were conducted via telephone and one via Skype. Interviews were audio-recorded and later transcribed. While an interview guide was developed (the REB approved interview guide is available in the Supplementary Material), it was used only as a probing tool; CTA interviewers rely more on training and knowledge of the macrocognition framework (see Table 2) upon which CTA is based [28], which we have used in previous studies [25], than upon structured interview questions. Macrocognition refers to the cognitive processes and functions that people use to make decisions in their natural settings and is the subject of extensive literature [28].

Participants were asked to walk through a specific case or two where they provided care for a patient living with cirrhosis. No identifiable patient information was collected, only age range and gender. Key probing questions were conducted in four sweeps. In sweep 1, the interviewer is looking to understand how liver disease management is set up in the clinic and how often the participant sees patients with cirrhosis. In sweep 2, the participant is asked to walk through a specific case from initial contact to follow-up, including who saw the patient, who was involved in care planning and coordination, how information was passed along, and how work was allocated. In sweep 3, the interviewer is framing the degree to which there is clarity in the roles or expectations of those involved in the patient's care, primarily with specialists and family physicians. And finally, in sweep 4 the interviewer tries to understand the consequences of the participant's choices by asking about counterfactuals—if the situation had been different, what would have changed. Participants were assigned a study number and all identifiable information was removed at the time of transcription.

### 2.3. Data Analysis

Audio recordings of the interviews were transcribed. The transcripts were then divided into sections and each section was coded by two CTA trained members of AMA-ACTT (led by LT), rotating by interview and mixing the pairings, using the macrocognition framework [30–32] (Table 2) and allowing for emergent findings. Analysis took place concurrently with data collection. Group analysis meetings were held with research team members (TB and LAG) and the CTA trained AMA-ACTT team members (including LT). These meetings involved coding review and attending to any disagreements, which were resolved by group consensus. We ensured that either the interviewer or the note taker of the interview under analysis was present, in order to clarify details, explain artefacts collected, and set the context of the interview and the participant's care setting. Using the coded data, the team then built a shared comprehension of how each participant carries out each macrocognitive function in their approach to cirrhosis care. This process took place via group discussion and the review of coded data, as noted above, so that any discrepancies could be resolved through group discussion and consensus. The team then reviewed all the narrative summaries of the macrocognitive functions, plus emergent findings, to build a narrative description of the participant's mental model of cirrhosis care. Finally, contrasts and comparisons across participants were compiled and categorized. Member-checking is not ordinarily part of CTA; though we have done it in prior CTA work [25], we did not in this case.

## 3. Results

Most of the family physicians did not have rich internalized mental models of cirrhosis care, but rather what we termed “knowledge on demand” or “experts in the moment” models. They would research, read, and access online resources or rely heavily on established relationships with specialists to become knowledgeable about cirrhosis when they were currently treating someone with the condition. However, due to the infrequency of treating those living with cirrhosis, the nature of their work, and its demands, they could not maintain expertise about cirrhosis (Table 3; Illustrative Quotations: 1.1). This resulted in the need to rebuild their mental models each time they saw a new patient living with cirrhosis.

There were individual differences in what family physicians included in the scope of their mental models. Some considered the whole patient, including any socioeconomic barriers challenging their health management, while others focused only on what they saw as primary care concerns and left cirrhosis-specific care to specialists (Table 3, 1.2).

There was consensus amongst family physicians that they lacked a structured process for coordinating care for those living with cirrhosis. The lack of a formal system for planning and prevention, as well as the necessity to rebuild knowledge each time they cared for a patient with cirrhosis, meant that many family physicians found the process effortful in nature. As a result, most developed reactive, patient-need-focused rather than proactive system-of-care mental models. This meant that physicians were dependent on who they knew in the specialist arena to access information and resources. Without these relationships many family physicians, and the patients they treated, lacked streamlined or structured care, including clear roles in terms of who would care for whom, when, and where (Table 3, 1.3). Finally, the geographical location of the physicians and the patients they treated also resulted in limited resources and/or connections with specialist care (Table 3, 1.4).

The specialists we interviewed on the other hand had rich mental models of cirrhosis. This was not surprising as they specialize in working with this patient population. The specialists described how cirrhosis care needed complex coordination and yet the system lacked the ability to support it. We described their mental models as the “Swiss cheese” model— they held rich mental models of idyllic cirrhosis care but also recognized the holes (gaps) in the health care system that prevented this ideally, including the coordination and continuity of care (Table 3, 2.1).

While all specialists recognized the challenges or holes (gaps) in the system, whether they saw filling or addressing them as part of their role varied. Some focused on what they could do *around* the holes, addressing only what the patient presented with and holding a more task- or work-based mental model. Others felt responsible for addressing the holes by taking on areas outside of their usual realm of care work, particularly if they felt roles were not clear, or other providers or areas of the health care system were not addressing patient needs. Both approaches were seen as effortful, and often reactive—challenged by having to work within the constraints of the existing health system (Table 3, 2.2).

While our data on nurse practitioners is limited to two participants, we found they both held empowered, patient-centered, and context-bound mental models. While they considered what was important to the patient, advocated for the patient, and were accountable to the patient, their mental models were bounded within the specialty in which they worked: the decompensated stage of cirrhosis. Their mental models were like the “Swiss cheese” model of specialists, as they too held rich understandings of ideal cirrhosis care while recognizing the holes (gaps) in the system. However, they differed from specialists in terms of believing it was their responsibility to address those holes as much as possible. Nurse practitioners had a wide latitude to work within scope, meaning they could make decisions and had agency in how they cared for those living with cirrhosis. This allowed them time to build relationships and trust with patients, thus addressing, together with the patient, the health, and social needs of day-to-day living (Table 3, 3.1).

Nurse practitioners reported their own challenges within the provincial health system. They described how they were often excluded in correspondence regarding their patient's care, such as patient hospital admission and discharge communication or witnessing their referrals being sent back to specialists within their clinic rather than directly to them (Table 3, 3.2).

One of the macrocognitive functions emerged as especially prominent in our analysis: managing the unknown, unexpected, and irregular. It was prominent in the mental models of both primary and specialty physicians, imposing a great deal of cognitive workload in addressing cirrhosis care coordination in Alberta.

Participants described in both direct and indirect ways that managing cirrhosis care becomes an act of constantly managing the unknown, or as one family physician described it, managing “*the expected unexpected events*” (Table 3, 4.1). The factors attributed to this act of managing the unknown included the following: (a) the disease itself, which was complex and included side effects such as cognitive impairments (Table 3, 4.2); (b) the additional comorbidities and challenges beyond cirrhosis, such as unstable housing, addiction, and other social determinants of health (Table 3, 4.3); and (c) geographic location which limits access to family physicians and specialists. Specialists and family physicians offered potential solutions to working with the unknown or “expected unexpected” when providing cirrhosis care. These included flexible scheduling, using peer support, and having trusted pathways (Table 3, 4.4).

## 4. Discussion

This detailed analysis of provider mental models of cirrhosis care presents several important findings. The analysis reinforces the challenges of cirrhosis care across providers, including the complexity of the illness, socioeconomic barriers, the lack of care coordination, clarity in roles and responsibilities, and for family physicians in particular, comfort in treating patients they infrequently see in their practices [14, 16, 17]. As a novel finding, it clarifies the mental models held by providers of cirrhosis care and how they apply that knowledge. For instance, we are now aware that family physicians' lack of comfort or higher reliance on specialists for treating patients living with cirrhosis is linked to their need to rebuild their mental models each time they see a new patient. Any program that aims to address such issues must consider this rebuilding and thus the need for trusted information that is easily and quickly accessed.

Family physicians and specialists held mental models that included reactive and effortful approaches to cirrhosis care due to a lack of system structure, process, and clear guidelines or tools. These gaps impeded planning, prevention, and consistent treatment or management, causing providers to create workarounds within their understanding of how to manage cirrhosis care. This, in turn, impeded the ability to develop rich mental models of cirrhosis care and likely determined whether they formed a patient-centred or task-based mental model.

The lack of clarity in roles and responsibilities extended to conversations and care planning around the trajectory of the illness and preparing for end-of-life. While a focus on palliative care was not specific to the aim of this study, nor was it something that participants discussed specifically, participants did identify a need for health providers to have clear roles and responsibilities throughout the continuum of providing care for those with cirrhosis. Nurse practitioners, in particular, described having the time to engage in honest conversations with patients in terms of where they were in that continuum, what they could expect, and any changes that could occur depending on what actions the patient took. This finding is similar to other studies that have demonstrated that family physicians, in particular, have a difficult time knowing when to have these discussions, whose responsibility it is to have these conversations, and how to manage symptom management or ongoing medical interventions with palliative care [16–18]. In order to explore where palliative care fits within family physicians' mental models of cirrhosis care, our plan is to conduct a second study specifically looking at symptom management.

The uncertainty that accompanies both the illness itself and a portion of the population living with cirrhosis augments the likelihood of forming restricted and reactive mental models of cirrhosis care. Some of the providers we interviewed discussed accepting the unknown and unexpected as the expected, and structuring their care in a flexible way so that they could provide more patient-centred or preventative care. Creating system-level supports that coincide with such ideas may assist other providers to accept this uncertainty and manage the “expected unexpected”, for example, implementing colocated teams; early screening and prevention; tracking processes; planning and replanning; and deliberate feedback loops. It may also facilitate care planning conversations in order to actively care for patients and plan for quality of life throughout all stages of this illness, including end-of-life [16, 18].

What high-level implications do these learnings have for programs such as CCAB that aim to improve the quality of care in cirrhosis? The heavy dependence of family physicians on knowledge-on-demand [33] and the need to rebuild a mental model when providing care for a patient with cirrhosis, suggest that explicitly incorporating knowledge management [34] principles may improve the effectiveness of CCAB and similar programs. Tools such as the planned CCAB electronic medical record orders set for hospitalized patients and evidence/expert consensus-based, website-based care pathways (http://www.cirrhosiscare.ca) hold promise for improving the efficiency and feasibility for family physicians to access the right kind of knowledge in the right format for their task, in the context of their busy clinical work.

All providers conceptualized cirrhosis care in the current system as somewhat chaotic, requiring a great deal of management of unexpected and irregular events and catching of things that fall through the cracks. This imposes a high cognitive workload, contributing to errors and impairing patient safety [35]. This issue needs to be considered in efforts to improve cirrhosis care. It is not enough to build pathways that improve communication or track processes; the fundamental understanding of managing the unknown and the cognitive support required for people managing uncertainty and unpredictability in high needs populations necessitates our full attention as a foundation to any intervention development.

Both between and within specialty and primary care, providers do not have a shared mental model of the boundaries of cirrhosis care. Family physicians differ in whether they believe cirrhosis-related issues “belong” to them or the specialist, specialists differ in whether they assume responsibility for noncirrhosis issues, and all physicians differ in whether they have a task-based or relationship-based model, that is, whether they address only medical issues or include social challenges patients face. Progress toward a more shared mental model, “getting everyone on the same page,” will likely require a dialog process amongst key stakeholders.

This study is limited by our sample. Though it is diverse, we do not know how closely we approached saturation. It is possible that additional interviews would yield incremental refinements, particularly by including more nurse practitioners in both specialty and primary care contexts. The strengths of this study are the use of CTA, a well-developed formal method of deeply understanding knowledge work, and the achievement of our benchmark for success: the delivery of actionable understanding of major features that can advance the aim of improving coordination and transitions amongst cirrhosis providers.

## 5. Conclusions

Our findings suggest that improving the coordination of cirrhosis care in Alberta will require more than improving communication, disseminating information, and addressing the logistics of consultation. The learnings from this work and the steps forward are likely to have relevance beyond Alberta and beyond liver cirrhosis. Similar gaps in coordination are anticipated across a variety of geographic locations and across chronic diseases that are not considered “garden variety” conditions for primary care physicians. A systematic approach that uses knowledge management to support the mental model that arises naturally in the family medicine context and that addresses cognitive workload reduction will enhance success. A province-wide dissemination and implementation plan that is specifically targeted at identified differences in mental models, at identifying where people are not on the same page, and what major value judgments (e.g., addressing social needs) need consensus development, also seems called for.

## Figures and Tables

**Table 1:** Participant demographics.

*N* = 19	Family physicians	Specialists	Nurse practitioners
Self-identified gender			
Woman	2	3	2
Man	6	6	

Age			
30–39 years old	2	2	
40–49 years old	5	3	
50–59 years old	1	3	2
60–69 years old		1	

Place of medical education			
In Canada	4	6	2
Outside of Canada	4	2	
Both		1	

Years practicing			
Under 10 years	2	2	
10–19	3	4	
20–29	2	2	1
30–39	1	1	1

Geographic location			
Urban	3	8	2
Rural	5	1	

**Table 2:** Macrocognition framework.

Function	Description
Sensemaking and learning (SL)	(i) Deliberate attempt to find coherent situational understanding
	(ii) Modifying a mental model or generating a new one
	(iii) Includes sense giving (presenting an understanding to others to adopt)
Decision making (DM)	(i) Decisions in, or about, patient care and administrative processes
Planning and replanning (PL)	(i) Shaping or reshaping patient care or administrative processes
Monitoring and problem detection (MD)	(i) Tracking the progress or outcomes of patient care or administrative processes
	(ii) Planned, ad hoc (“noticing”), formal (data collection), or informal
Managing the unknown, unclear, unexpected, and irregular (MU)	(i) Planned or anticipatory (contingencies, fallbacks)
	(ii) Evaluating/estimating risks
	(iii) Unplanned, “scrambling”
Coordinating (CO)	(i) Any activity that helps synchronize 2 or more individuals in a patient care or administrative process, especially transmitting information or expectations
	(ii) Maintenance of “common ground,” shared expectations/understanding/mental models of processes

**Table 3:** Illustrative quotations from data generated by CTA interviews with family physicians (FPs), specialists (SPs), and nurse practitioners (NPs).

Illustrative quotations	
1.0 *Family physicians' mental models*	
1.1 Have to rebuild their mental models—practicing as “experts in the moment” and requiring “knowledge on demand”	“Yes, I think this is quite typical of primary care,… my knowledge and skill has been upscaled the longer I've looked after him. I've learnt from the helpful letters from the liver clinic…. Sadly, when I'll no longer look after him, I'm sure I'll gradually descale again, but again, with the myriad of other conditions I'll become an expert in another area, and another area….” (FP3)
	“Enough that it's a problem, but not enough that maybe we're good at it [family physicians seeing patients with cirrhosis]. It's not like heart failure or pneumonia - pretty garden variety stuff you see all the time, but when the cirrhotics come in, especially the decompensated ones, they're very sick and there's a lot of intricacies to think about.” (FP2)
	“All of these things are sort of foreseeable in various ways, shapes or forms and yet every single time it's like you're reinventing the wheel.” (FP4)

1.2 Vary in what they consider to be the primary care scope for managing patients with cirrhosis	“I'm trying to deal with the alcohol and I'm trying to help with some of the barriers as to why he's drinking again and then also trying to deal with some of the... he doesn't have any access to money, and I'm trying to get him into some programs, you know, get him established.” (FP5)
	“Yes, like I mean, I was mostly leaving it up to the hepatology clinic.” (FP6)
	“…very few people will ask him about depression, how's your mood doing with all of this? How's your relationship with your wife? What's happening with your children? … trying to be, you know, as it says on our label - family physician, so therefore involving the family with that as well”. (FP3)

1.3 Depend upon relationships to seek guidance	“It makes me realize reflectively just how complicated this thing is…. What it shows is a lot of this complex care is about relationships… I see a really strong team who looks after this patient, and sadly all it takes is for one of those members in that team to change for a period of time and that patient ends up becoming an admission, which could've been avoided.” (FP3)
	“It's all we could do was (a) discover he was admitted, (b) call to see if he was still admitted because of course the time delay there, and then by the time that had happened they were already on discharge planning or had already discharged him…well wouldn't it be nice if we…could have collaborated a little bit more and tried to find a bit of an overall solution to this instead of just playing the admission/discharge deterioration game over, and over again”. (FP4)

1.4 Experience geographic location as major barrier	“The city is about two hours away and a lot of people don't want to get themselves involved in traveling, so there are so many challenges when you're referring to places in our area.” (FP1)
	“Well, you know, it's probably very difficult for them (the patients) because these people are traveling a great distance… the travel is a bit of a barrier. We don't have access to the specialist that would do our cirrhosis care.” (FP2)

2.0 *Specialists' mental models*	
2.1 Have rich mental models of cirrhosis care but are well aware of the gaps—“swiss cheese model”	“I think the way our system is structured now is very poor…like these cirrhotic patients, many of them they're not going to just have cirrhosis. A lot of them are going to have diabetes, they're going to have hypertension, they're going to have heart failure, they're going to have all sorts of other diagnostic problems, and I think the subspecialty model has failed to address that.” (SP7)
	Well I work with other physicians in the hospital, but it's not a team, like I see my own patients and they're my responsibility. I don't have anybody like working with me per se… I don't have a nurse, I don't have a dietician or a pharmacist that works with me, it's just me…if a patient is really sick it's me...having to be alert as to whether … am I missing anything. It would be nice to have backup or help. (SP3)
	We sometimes don't carefully delineate, hey you're going to be responsible for, you know, the colon cancers, you know, the rectals and the PAP tests and the mammograms, and, we're going to take care of the cardiovascular arresting, who's going to manage the blood pressure. (SP1)

2.2 Vary in what they see their role is in filling the gaps in cirrhosis care	“I've always considered myself to be like their primary liver specialist,…any problems that had to do with their liver, so cancer, bleeding, whatever…that would be my duty to take care of… if a patient thinks that they're swelling up and they need a paracentesis I expect them to call me not their family doctor … I'm the bridge toward referral to transplant, my level of involvement depends on how sick they are.” (SP4)
	“Most of my patients in my clinic practice do have a family physician… if they don't have a family physician and the diagnosis is serious enough I will actually follow them until I've sorted it out…I would volunteer to keep an eye on them over the next few months until we... found another family doctor or sorted out who's going to look after them.” (SP7)
	“…my letters, they're extremely long and detailed… a kind of laundry list of things that they (family physicians) would hopefully check off…when that doesn't happen, which is often the case because a lot of these patients end up actually kind of being not attached… then it kind of falls back to me… I don't get a letter back from the family doctor saying they're doing anything. I see them (the patient) and nothing's been done, nothing's been checked, so it's not the majority but it's a sizeable minority of patients that are pretty uncared for in general... it's not really clear if there's someone actually managing their care overall.” (SP9)

3.0 *Nurse practitioners' mental models*	
3.1 Rich mental models—similar to “swiss cheese” model but taking responsibility for the gaps	“…I think we've got a long way to go in terms of understanding what our patients understand. We don't often ask. We have to take the time to explain and to educate and to ensure that they [patients] understand, and give them an opportunity to ask those questions.” (NP1)
	“…I think it's a real bonus to patients because I do have the time a physician may or may not have to spend with that patient, and actually get to know them and hear about what's going on in their lives, and what matters to them, and what challenges they're having, it's not just specifically - I'm here to deal with your medical issues and we're done so see you later.” (NP2)

3.2 Challenges facing nurse practitioners in coordinating cirrhosis care	“…there are those physicians that I will send a referral to and they will return the referral back to one of the hepatologists, so the letter goes back to them, not to me, even though I was the person that sent the referral, it's challenging… “(NP2)
	“We're rarely notified that they're [patient] in hospital. We're inconsistently copied on the discharge summaries. So we don't often know when there's a gap in care until it's too late.” (NP1)
4.0 *Prominent category: managing the unknown, unexpected, and irregular*	
4.1 The expected unexpected	“… the recurrent unexpected happened in terms of, you know, unpredictable Emergency room visits, so any particular day was hard to predict, but globally recurrent visits to the Emergency room, recurrent visits to the clinic, recurrent sort of stretches where [patient] wouldn't go to the pharmacy because of med compliance etc., so those are sort of the expected unexpected events.” (FP4)

4.2 Complexity of cirrhosis care	“…he had episodes of hepatic encephalopathy, so that's what made his care management more challenging was the cognitive aspect of that. …He would lose housing frequently. He would go in and out of Emergency because of his thinking, his med compliances, edema, so just chasing him down wherever he went and trying to work out a proactive plan for that, it never really seemed to happen. We were always chasing our tail it seemed. (FP4)
	“…these patients in advanced cirrhosis are challenging patients, and there's social issues, there's medication issues, there's blood tests to be done, and there's all sorts of other issues.” (SP7)

4.3 Socioeconomic instability	“Apart from that I also hope that as family physicians we would focus a little more on other social determinants for the patient because many of them have real issues. We know housing issues, money issues, addictions issues. Some of them need to go to rehab.” (FP7)
	“The trouble is a lot of these people get labelled, and society is very judgemental about them and doesn't…really care about them.” (SP7)

4.4. Potential solutions	“I just wish I had an understanding for … what to do… I would want some sort of pathway that I know I can rock solidly rely on.” (FP5)
	“I think that's where peer support workers could be really useful is helping people who are so reticent to go for their screening or for either liver cancer or esophageal varices because it's a really frightening thing.” (SP2)
	“Flexibility and scheduling…have like a half-day a month where no one needs a specific hard appointment to be seen… you know, last Friday of the month is marginalized population day…” (FP4)

## Data Availability

The qualitative data used to support the findings of this study, beyond use of selected quotations, are restricted by the Research Ethics Board at the University of Alberta in order to protect participant privacy.

## Supplementary Materials

Supplementary Materialsupdated title to match the manuscript Supplementary File 1: REB Approved Interview Guide/Probes. Supplementary File 2: SRQR Checklist.
