# Supplementary material for: Eliciting and Understanding Primary Care and Specialist Mental Models of Cirrhosis Care: A Cognitive Task Analysis Study
Source: Can J Gastroenterol Hepatol. 2021 Jun 15;2021:5582297. doi: 10.1155/2021/5582297 (PMC8219466; doi:10.1155/2021/5582297)
Supplement: Supplementary Materialsupdated title to match the manuscript — Supplementary File 1: REB Approved Interview Guide/Probes. Supplementary File 2: SRQR Checklist. [file 5582297.f1.zip › Suppl File 1_Interview Guide_210521.docx]

**Cirrhosis Care Alberta (CCAB) CTA Research Sub-Study**

**Understanding primary and specialty care physician’s mental models**

Physician and Nurse Practitioner Interview Probes

These probes should serve only as a general interview guide. They are intended for use as a memory aid by team members trained in Cognitive Task Analysis. They are not to be asked verbatim or in order, nor are all necessarily expected. Depending on the interviewee and the information gained in other interviews, other probes may arise.

Introduce and frame the question for either the family physician, specialist physician or nurse practitioner:

We are seeking to understand the thinking and decision-making processes that take place when you care for and coordinate care of patients living with cirrhosis. We would like to know more about your role and thinking within this process. We will ask you some questions about how liver disease is managed in your clinic and then ask you to run through a specific case or two where you cared for a patient living with cirrhosis, preferably from early (compensated) to decompensated disease (where complications like ascites or hepatic encephalopathy have developed), and possibly to the last few months of life. Do not provide name or other identifying information of the patient but only approximate age and gender.

Sweep 1: structure and function. “Describe how liver disease management is set up in your clinic.” Obtain information about:

- How often they see patients with cirrhosis
- What technology or reports they use to provide care, how used, (e.g., formal complex care plan)
- Who is involved, in what aspects & roles – both officially and unofficially – within clinic and without (relationships between specialty & primary care & patients)

Sweep 2: walk through the process, from initial contact (by patient or team) to follow-up.

- Recall a specific case where you cared for a patient with cirrhosis from early (compensated) to decompensated illness, and possibly to end stage (last few months of life).
- Who saw them when, who does what care and coordination?
- How was care planned, and who was involved?
- How was information passed among team, activities done offline or out of visit times?
- How was the work allocated? Who made the decisions, and who had voice in them?
- How needs are determined, gaps caught, where things fall through or go astray?

Sweep 3: Clarity or roles and expectations

- Role clarity - How do you know what your role is?
- What do you perceive to be your responsibility in the care process?
- What do you believe the specialist/FP/NP should be doing? How do you believe they approach their work in the care process? Why do you think they do things the way they do?
- What is the working relationship like between the primary and specialist teams?
- What are the expectations of patients?
- Do you have any ideas on how to improve role clarity between primary and specialty care as well as between physicians, nurse practitioners and patients?
- How do you know when it’s working well, and when it’s not? (probe for specific examples, the who/how as well as what)

Sweep 4: Counterfactuals - Set of questions designed to understand the consequences of FP’s /specialist’s/NP’s choices. What if you had advised x, y, or z instead? What if (cue x) had been different? When might your recommendations have been different?

- What if (X) has been different?
- X could be:
  - Information asked (what have you done when you didn’t have the information you needed or needed clarity?)
  - Expectations (what if patient/specialty or primary care physician/NP had not done what you expected?)

Closing

- Broader closing question:
  - What resources or processes would you find helpful when managing treatment for patients living with cirrhosis?
- Do you have any questions for us? Or anything further you would like to add?
